# The Role of Wnt/β-Catenin Signaling in Enterocyte Turnover during Methotrexate-Induced Intestinal Mucositis in a Rat

**DOI:** 10.1371/journal.pone.0110675

**Published:** 2014-11-06

**Authors:** Igor Sukhotnik, Tatiana Geyer, Yulia Pollak, Jorge G. Mogilner, Arnold G. Coran, Drora Berkowitz

**Affiliations:** 1 The Bruce Rappaport Faculty of Medicine, Technion-Israel Institute of Technology, Laboratory of intestinal adaptation and recovery, Dept of Pediatric Surgery, Bnai Zion Medical Center, Haifa, Israel; 2 The Bruce Rappaport Faculty of Medicine, Technion-Israel Institute of Technology, Laboratory of intestinal adaptation and recovery, Haifa, Israel; 3 The Bruce Rappaport Faculty of Medicine, Dept of Pediatric Surgery, Bnai Zion Medical Center, Haifa, Israel; 4 Section of Pediatric Surgery C.S. Mott Children's Hospital and University of Michigan Medical School, Ann Arbor, Michigan, United States of America; 5 The Bruce Rappaport Faculty of Medicine, Technion-Israel Institute of Technology, Dept of Pediatric Gastroenterology, Bnai Zion Medical Center, Haifa, Israel; Van Andel Institute, United States of America

## Abstract

**Background/Aims:**

Intestinal mucositis is a common side-effect in patients who receive aggressive chemotherapy. The Wnt signaling pathway is critical for establishing and maintaining the proliferative compartment of the intestine. In the present study, we tested whether Wnt/β-catenin signaling is involved in methotrexate (MTX)-induced intestinal damage in a rat model.

**Methods:**

Non-pretreated and pretreated with MTX Caco-2 cells were evaluated for cell proliferation and apoptosis using FACS analysis. Adult rats were divided into three experimental groups: Control rats; MTX-2 animals were treated with a single dose of MTX given IP and were sacrificed on day 2, and MTX-4 rats were treated with MTX similar to group B and were sacrificed on day 4. Intestinal mucosal damage, mucosal structural changes, enterocyte proliferation, and enterocyte apoptosis were measured at sacrifice. Real Time PCR and Western blot was used to determine the level of Wnt/β-catenin related genes and protein expression.

**Results:**

In the vitro experiment, treatment with MTX resulted in marked decrease in early cell proliferation rates following by a 17-fold increase in late cell proliferation rates compared to early proliferation. Treatment with MTX resulted in a significant increase in early and late apoptosis compared to Caco-2 untreated cells. In the vivo experiment, MTX-2 and MTX-4 rats demonstrated intestinal mucosal hypoplasia. MTX-2 rats demonstrated a significant decrease in FRZ-2, Wnt 3A Wnt 5A, β-catenin, c-myc mRNA expression and a significant decrease in β-catenin and Akt protein levels compared to control animals. Four days following MTX administration, rats demonstrated a trend toward a restoration of Wnt/β-catenin signaling especially in ileum.

**Conclusions:**

Wnt/β-catenin signaling is involved in enterocyte turnover during MTX-induced intestinal mucositis in a rat.

## Introduction

Oral and gastrointestinal mucositis is a frequent dose-limiting and costly side effect of cytoreductive cancer chemotherapy which affects more than three-fourths of patients [1], [2]. Mucositis represents a significant burden to patients and caregivers. It leads to dose reduction or prevention of continuation of selected cancer therapies, increases healthcare cost, prolongs hospital stay, increases re-admission rates, compromises patients' nutritional status, impairs patients' quality of life, and is occasionally fatal [3]. Despite advances in the understanding of mucositis over recent years, the underlying mechanisms of the condition are poorly understood. Similar to the tumor target, the DNA of proliferating intestinal stem cells undergoes strand breaks, resulting in direct cellular injury [4]. In addition, chemotherapy may exert cell-damaging or a cell-destroying effect through the generation of reactive oxygen species [5], or through enzymatic or transcription factors (NF-κB) which leads to up regulation of genes responsible for production of proinflammatory cytokines TNF-α, IL-1β and IL-6^2^. This leads to tissue injury and apoptosis [6].

The integrity of the gastrointestinal epithelia is highly dependent on resident self-renewing intestinal stem cells (ISCs), which makes them vulnerable to chemical insults (as from chemotherapy agents) compromising the repopulating capacity of the epithelial stem cell compartment. The signals that initiate ISCs invasion are not well understood, but there is increasing evidence that extracellular physical signals play an important role. Growing evidence suggests that Wnt/β-catenin signaling plays a pivotal role in both embryonic development and homeostatic self-renewal of stem cells, including gastrointestinal and oral mucosa [7]. The main output of Wnt signaling is to regulate the stability of β-catenin. In the absence of Wnt, β-catenin is associated with the multiprotein β-catenin destruction complex that consists of Axin, adenomatous polyposis coli (APC) protein, and glycogen synthase kinase 3 (GSK3). In this complex, is constitutively phosphorylated by GSK3, which triggers β-catenin degradation through the ubiquitin-proteasome pathway. The Wnt signal is received by Frizzled receptor and the low-density lipoprotein receptor related protein 5/6 (LRP5/6). Wnt binding induces phosphorylation of LRP5/6, and phosphorylated LRP5/6 binds to Axin, which leads to the dissociation of the β-catenin destruction complex. Stabilized β-catenin enters the nucleus, binds to the TCF transcription factors and initiates transcription of Wnt responsive genes [8]. There is a gradient of β-catenin expression along the intestinal crypt axis with the highest levels at the crypt bottom [9]. In addition, colorectal cancers show a heterogeneous subcellular pattern of β-catenin accumulation. However, it remains unclear whether different levels of Wnt signaling exert distinct roles in the intestinal epithelium. It is unclear whether Wnt/β-catenin signaling regulates intestinal induction directly, cell-autonomously, or by controlling signaling from surrounding cells, non-cell-autonomously.

The purpose of this study was to evaluate the role of Wnt/β-catenin signaling during recovery from chemotherapy-induced mucositis in a rat model.

## Materials and Methods

### Cell cultures and treatment

Caco-2 cells (ATCC-R-HTB 37TM were purchased from ATCC (Israeli distributor BIOLOGICAL INDUSTRIE ISRAEL BEIT HAEMEK LTD). Caco2 cells were grown in Dulbecco's modified Eagle's medium supplemented with 2 mM glutamine and 10% fetal calf serum in a 5% CO_2_ humidified atmosphere at 37°C. Monolayers were subcultured every 7 days by trypsinization with 0.1% trypsin and 0.9 mmol/liter EDTA in Ca^2+^/Mg^2+^-free phosphate-buffered saline.

### Cell proliferation

Rapid detection of S-phase cells was determined by FACS brand flow cytometry using anti-bromodeoxiuridine(BrdU) antibody. Briefly, BrdU labeling reagent (diluted 1∶100) was added directly to the culture medium to achieve a final concentration of 10 µM. For direct immunofluorescence staining, 20 µL of Anti-BrdU FITC (Fluorescein isothiocyanate) per 10^6^ cells was added and the suspension was incubated for 30 minutes at room temperature. The cells were centrifuged at 500x*g* for 5 minutes and re-suspended in 1 mL of 1X PBS containing 5 µg/ml of propidium iodide. BrdU incorporation was analyzed on a FACS brand flow cytometer with laser excitation at 488 nm. Experiments were performed in duplicate and repeated at least three times.

### Cell apoptosis

Apoptosis was assessed using FACS analysis at 24 h by annexin staining and at 48 h by propidium iodide (PI). For annexin staining, cells were trypsinised and re-suspended in 1 ml annexin-binding buffer (BD Pharmingen, BD Biosciences, San Jose, Ca) to which was added 5 µl Annexin V-FITC (2.5 µg/ml; BD Pharmingen). After incubation in the dark at room temperature for 15 min, 50 µl PI (50 µg/ml; Sigma, Israel) was added to discriminate dead cells and the samples were analyzed on a FACS Caliber flow cytometer (Becton Dickinson, Franklin Lakes, NJ).

### Real Time PCR

Caco-2 cells were treated with MTX in dose 250 nM for 48 h in serum-free medium. RNA was isolated with TRIzol (Invitrigen) following the manufacturer's instructions. RNA was eluted in 200 µl RNase free water and RNA concentration measured using NanoDrop ND-1000 spectrophotometer (Thermo Fisher Scientific, Roskilde, Denmark).

Total RNA (1 µg) was reverse-transcribed using the PrimeScript RT reagent Kit Supermix for RT-qPCR (#RR037Q, Takara Bio Inc., Japan) in a total volume of 20 µl according to the manufacturer's instructions. The Supermix contained both oligo dT and random primers to obtain a maximum number of cDNA transcripts. The reaction mixture was incubated at 37°C for 15 min for reverse transcription, and finally at 85°C for 5 sec for reverse transcriptase inactivation. QPCR was performed with 4 genes (GenBank: Wnt3: NM030753, c-Myc: NM002467, CTNNB1: NM 001098209.1 and GAPDH: NM002046) using primer pairs were synthesized by Syntezza Bioscience ltd. Israel. Primer sequences were also cross-checked using web-based tool in-silico at the human genome browser at UCSC against gene and genomic targets.

### Animals

The protocol was approved by the Animal Research Committee of Rappaport Faculty of Medicine, Technion (Haifa, Israel) in compliance with the guidelines established by the “Guide for the Care and Use of Laboratory Animals”. Male Sprague-Dawley rats weighing 280–320 g were used for the experiment. The animals underwent a 3-day period of acclimatization under standardized conditions (12-hour day/night rhythm, temperature 22°C and 55% relative humidity) and were fed a standard rat chow.

### Study design

Animals were divided randomly into three experimental groups of 10 rats each. Group A (CONTR) rats were treated with a single intraperitoneal (IP) injection of normal saline (1 ml). Group B (MTX-2) animals were treated with a single dose (20 µg/kg) of methotrexate given IP and were sacrificed on day 2, and group C (MTX-4) rats were treated with MTX similar to group B and were sacrificed on day 4.

### Intestinal mucosal parameters

Two or four days following MTX injection, rats were anesthetized with IP Phenobarbital (75 mg/kg) and sacrificed by open pneumothorax. The small bowel was rapidly removed, rinsed with cold isotonic saline and divided into two segments: proximal jejunum (10 cm from Treitz ligament) and terminal ileum (10 cm proximal to ileocecal junction). Each segment was weighed, opened longitudinally and the mucosa was scraped from the underlying tissue using a spatula (Sigma, Israel). Mucosal samples were homogenized with TRIzol reagent (Gibco BRL, USA). DNA and protein were extracted by the method of Chomczynski [10].

### Histologic examination

After fixing in 4% buffered formalin, perpendicular sections from proximal jejunum and distal ileum were processed into standard paraffin blocks. Five-micron tissue slices were stained with hematoxylin-eosin. These sections were studied microscopically using a micrometer eyepiece. The villus height and crypt depth for each specimen were measured using an objective mounted micrometer (100xmagnification) and an optical microscope (10×100 magnification). Villus height and crypt depth data were from eight rats, and each measurement consisted of the mean of five villi and crypts.

The mucosal damage of the small bowel was graded using an intestinal injury score as described by Kesik et al [11]. The following parameters were investigated in the jejunum and ileum: (1) degeneration of surface and crypt epithelium, (2) degeneration of villus structure, vacuolization in the surface epithelium (3) inflammatory cell infiltration, and bleeding and edema in the lamina propria. For each parameter, a score was given using a semiquantitative scale as follows: 0 =  none, 1 =  mild, 2 = moderate, 3 =  severe, giving a maximum possible score of 9 for each intestinal region.

#### Crypt Cell Proliferation and Enterocyte Apoptosis

Rats were injected with standard 5-bromodeoxyuridine (5-BrdU) labeling reagent (Zymed Lab, Inc, CA) at a dose of 1 ml per 100 g body weight, two hours before sacrifice. Tissue slices (5 µm) were de-paraffinized with xylene, rehydrated with graded alcohol, and stained with a biotinylated monoclonal anti-BrdU antibody system using BrdU Staining Kit (Zymed Lab, Inc, CA). An index of proliferation was determined as the ratio of crypt cells staining positively for BrdU per 10 crypts.

Additional 5 µm thick sections were prepared to establish the degree of enterocyte apoptosis. Immunohistochemistry for Caspase-3 (Caspase-3 cleaved concentrated polyclonal antibody; dilution 1∶100; Biocare Medical, Walnut Creek, CA) was performed to identify apoptotic cells using a combination of streptovidin-biotin-peroxidase method according to manufacturers' protocols. Enterocyte apoptosis was expressed as the total number of apoptotic cells along this axis per 10 villi and 100 crypts. In some cases a more detailed analysis of the location of apoptosis was performed using previously established techniques. For this, apoptosis along the villi was differentiated between the lower one-third of the villi (lateral villi) and upper one-third of the villi (villi tips). Apoptosis was recorded as the number of apoptotic cells per 10 villi. A qualified pathologist blinded as to the source of intestinal tissue performed all measurements.

#### Real-time PCR

RNA was isolated using Trizol (Invitrogen) reagent according to the manufacturer's instructions, and quantification of RNA was performed using 260/280 nm spectrophotometry. The method was extended using reverse transcriptase (PrimeScript RT reagent Kit Takara, Japan) to convert 50000 ng of total RNA into complementary DNA (cDNA) which was then amplified by PCR-Thermal Cycler (2720 Thermal Cycler, ABI, Israel). Expression of Wnt/β-cathenin pathway related genes (Frz2, Wnt 3A, Wnt 5A, β-cathenin, C-myc, Cyclin D1) was determined by quantitative real-time PCR ABI-PRISM 7500 (Applied Biosystems, foster City, CA) on cDNA samples using Cyber Green Master Mix (Takara, Japan) with the exception of tmplate and primers and normalized to three housekeeping genes. All primerswere synthetized by ITD, Belgium.

#### Western blotting

Tissue was homogenized in RIPA lysis buffer containing 50 mM Tris–HCl (pH 7.4), 150 mM NaCl, 1% NP-40, 2 mM EDTA, supplemented with a cocktail of protease and phosphatase inhibitors. Protein concentrations were determined by Bradford reagent according to the manufacturer's instructions. Samples containing equal amounts of total protein (30 µg) were desolved by SDS-PAGE under reducing conditions. After electrophoresis, proteins were transferred to a PVDF membrane and probed with various primary antibodies to anti-β-catenin antibody (1∶1000 dilution, sc-8312 antibody MW 56-62 non phosphorylated), anti-Akt 1/2/3 antibody (1∶500 dilution, sc-59737 antibody non phosphorylated), anti-GSK3 (1∶5000 dilution, ab18893) and anti-actin antibody (1∶5000 dilution, A5316). Horseradish peroxidase-conjugated secondary antibody was purchased from Jackson ImmunoResearch Laboratories Inc. (West Grove, PA) and an enhanced chemiluminescent substrate from Biological Industries (Kibbutz Beth HaEmek, Israel). The optical density of the specific protein bands was quantified by using Totalab densitometer analysis software.

## Results

### Effect of MTX on Caco-2 cell proliferation and apoptosis

**Figure 1:**
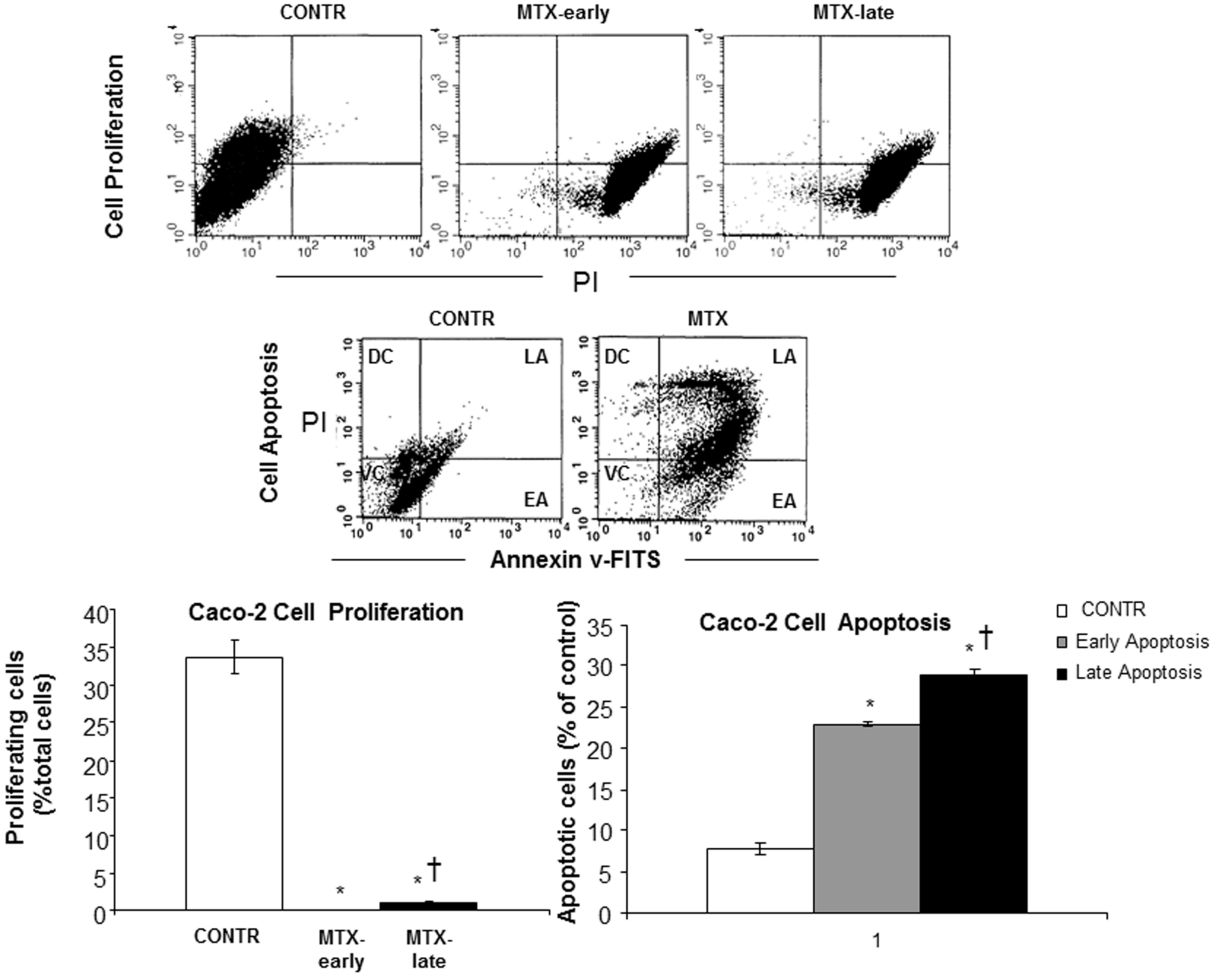
The effect of MTX on Caco-2 cells proliferation and apoptosis. Two color FACS systems analysis using anti-BrdU and propidium iodide (PI) to determine cell proliferation. FITC Annexin V assay for identifying cells that are undergoing apoptosis. MTX-methotrexate., EA-early apoptosis, LA-late apoptosis, VC-viable cells, DC-dead cells, PI- propidium iodide. Values are mean ± SEM. * P<0.05 MTX-early vs non-treated Caco-2 cells, †P<0.05 MTX-late vs MTX- early.

As demonstrated in Figure 1, treatment with MTX resulted in a 614-fold decrease in early cell proliferation rates over corresponding control cells treated with vehicle alone (p<0.001), followed by a 17-fold increase in late cell proliferation rates (p<0.001) compared to early proliferation; however, late proliferation was still 36-fold lower than the corresponding control cells treated with vehicle alone (p<0.001). Treatment with MTX resulted in a significant six-fold increase in early apoptosis (p<0.001) and four-fold increase in late apoptosis (p<0.001) compared to Caco-2 untreated cells.

### Expression of Wnt/β-catenin related genes in Caco-2 cells

Two days after exposure to MTX, Caco-2 cells demonstrated a significantly higher β-catenin mRNA levels (51%, p<0.001) and significantly lower c-Myc mRNA levels (65%, p<0.001) over corresponding control cells with vehicle alone (Table 1). Treatment with MTX did not change significantly Wnt3 mRNA levels compared with non-treated cells.

**Table 1:** Effect of methotrexate on Wnt/β-catenin related genes in Caco-2 cell line.

Cell Line	β-catenin	Wnt3	c-Myc
Caco-2 nontreated	1	1	1
Caco-2 treated with MTX	1.51±0.145 P<0.001	1.040±0.233 P-NS	0.349±0.009 P<0.001

### Animals

#### Final Body Weight

**Figure 2:**
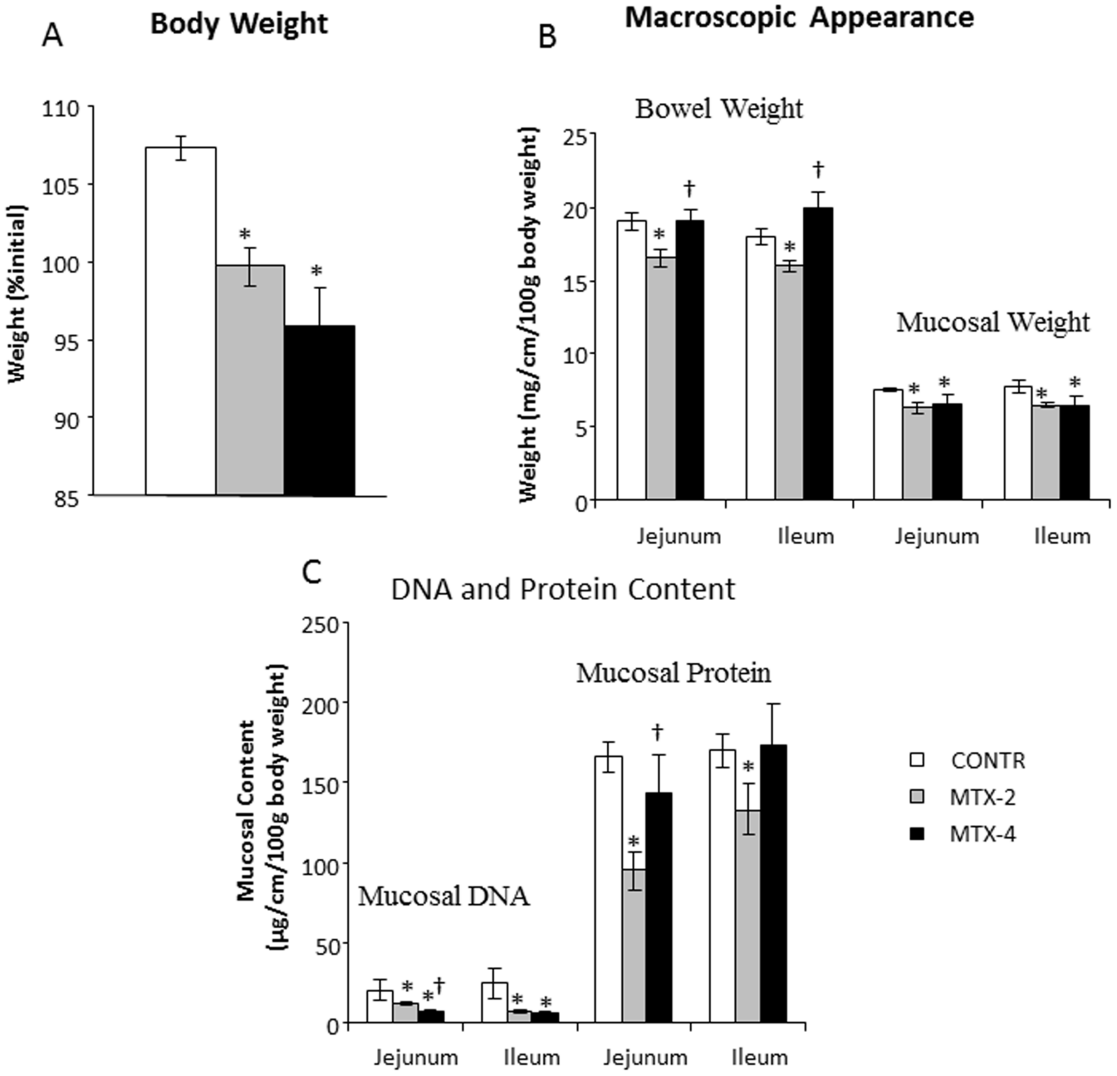
Effects of MTX on body weight changes (A), bowel and mucosal weight (B) and mucosal DNA and protein (C). Values are mean ± SEM. CONTR- control, MTX-methotrexate. * P<0.05 MTX-2 and MTX-4 vs CONTR rats, † P<0.05 MTX-4 vs MTX-2 rats.

MTX-2 (Group B) rats demonstrated a significantly lower final body weight compared to control animals (Group A) (100±1 vs 107±1%initial weight, p<0.05) (Figure 2A). MTX-4 rats demonstrated an additional decrease in final body weight compared to MTX-4 animals; however, this trend was not statistically significant.

#### Intestinal Mucosal Parameters

MTX-2 rats (Group B) demonstrated a significant decrease in overall bowel weight in jejunum (13%, p<0.05) and in ileum (11%, p<0.05), as well as in mucosal weight in jejunum (16%, p<0.05) and ileum (17%, p<0.05) compared to control animals (Figure 2B). Four days following MTX administration, MTX rats (Group C) demonstrated a significant increase in jejunal (15%, p<0.05) and ileal (25%, p<0.05) overall weight, as well as well as unchanged mucosal weight compared to MTX-2 counterparts. A significant decrease in mucosal DNA was observed in jejunum (two-fold decrease, p<0.005) and ileum (four-fold decrease, p<0.005) in MTX-2 rats (Group B) compared to control animals (Figure 2C). Additionally, MTX rats demonstrated a significant decrease in mucosal protein content in jejunum (43%, p<0.001) and ileum (22%, p<0.05) compared to control animals. Four days following MTX administration, MTX rats (Group C) had a significant increase in jejunal (50%, p<0.05) mucosal protein (and a trend in ileal mucosal protein) as well as unchanged mucosal DNA content compared to MTX-2 animals (Group B).

#### Microscopic changes

**Figure 3:**
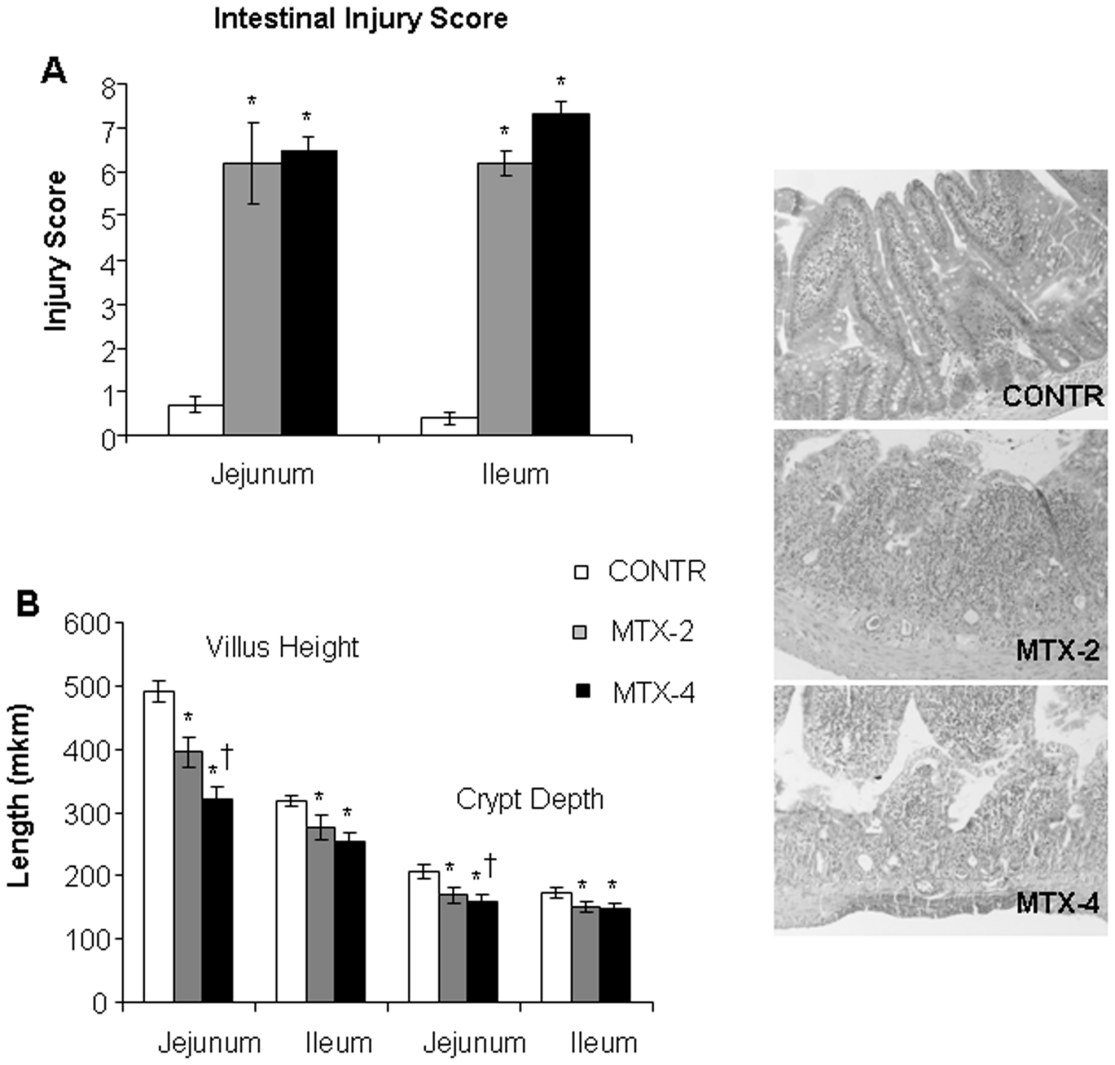
Grade of intestinal mucosal injury and microscopic intestinal appearance after administration of MTX. Values are mean ± SEM. CONTR- control, MTX-methotrexate. * P<0.05 MTX-2 and MTX-4 vs CONTR rats, † P<0.05 MTX-4 vs MTX-2 rats.

The degree of intestinal damage increased following MTX administration, as indicated by severe villous atrophy, epithelial flattening, significant loss of crypt architecture, signs of crypt remodeling, severe villous epithelial atrophy, degeneration and shortening of the villus length, and polymorphonuclear leukocyte infiltration in the lamina propria. Two days following methotrexate administration, MTX-rats (Group B) demonstrated a significant increase in intestinal damage score in jejunum (6.2±0.9 vs 0.7±0.02, p<0.001) and distal ileum (6.2±0.2 vs 0.4±0.16, p<0.001) compared to control animals (Figure 3A). MTX-4 rats (Group C) demonstrated a more significant damage in the distal ileum (7.3±0.3 vs 6.2±0.3, p<0.05) compared to MTX-2 animals (Group B).

Two days following MTX administration, MTX rats (Group B) demonstrated a significant decrease in jejunal (396±24 vs 491±13 µm, p<0.05) and ileal (277±19 vs 318±9 µm, p<0.05) villus height, as well as well as in jejunal (169±12 vs 206±11 µm, p<0.05) and ileal (151±8 vs 173±8 µm, p<0.05) crypt depth compared to control counterparts (Figure 3B). MTX-4 rats (Group C) demonstrated an additional decrease in jejunal villus height (321±20 vs 396±24 µm, p<0.05) compared to MTX-2 animals as well as a trend toward additional decrease in ileal villus height and crypt depth; however, this trend was not significantly significant.

#### Enterocyte proliferation and apoptosis

**Figure 4:**
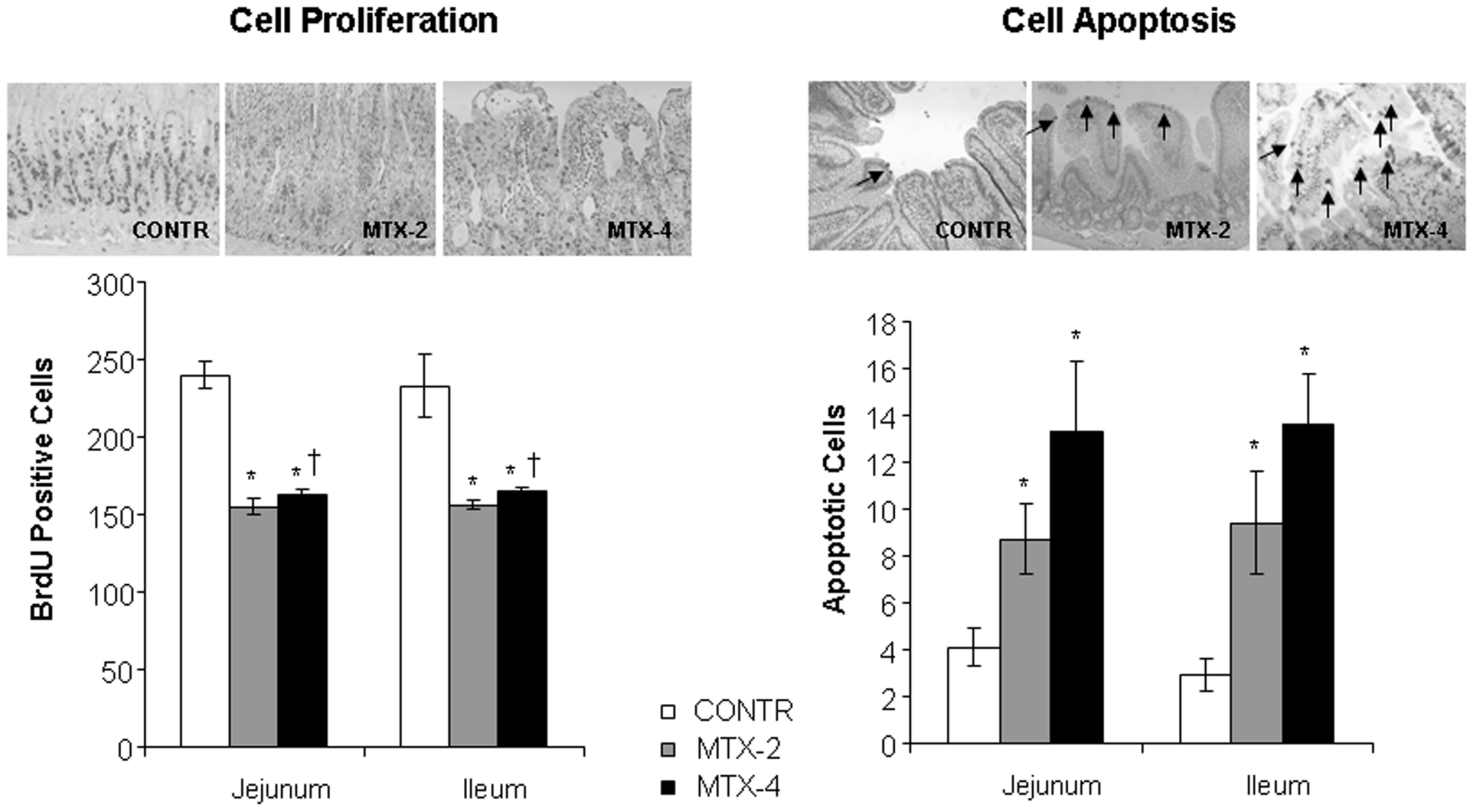
Effects of MTX on crypt cell proliferation (A) and apoptosis (B). The number of labeled cells in 10 well-oriented, longitudinal crypts per section from each rat was determined using light microscopy (B). The apoptotic index is expressed as the percentage of apoptotic cells per 10 villi. Values are mean ± SEM. CONTR- control, MTX-methotrexate. * P<0.05 MTX-2 and MTX-4 vs CONTR rats, † P<0.05 MTX-4 vs MTX-2 rats.

The proliferative zone of MTX-2 rats was only mildly affected, showing a slight shift upwards within the crypts. The proliferative zone in MTX-4 rats moved progressively upwards in the crypts toward the crypt-villus junction. MTX-2 rats (Group B) demonstrated a significant decrease in enterocyte proliferation index in jejunum (155±5 vs 240±9 BrdU positive cells/10 crypts, p<0.001) and ileum (156±3 vs 233±20 BrdU positive cells/10 crypts, p<0.001) compared to control animals that remained unchanged after 4 days (Group C) (Figure 4A).

Significantly greater numbers of apoptotic cells appeared in the villi of jejunum (two-fold increase, p<0.05) and ileum (three-fold increase, p<0.05) of MTX-2 rats (Group B) compared to control animals (Figure 4B). Although MTX-4 (Group C) rats showed a trend toward an additional increase in cell apotosis in jejunum and ileum compared to MTX-2 animals (Group B), this trend did not achieve statistical significance.

#### Expression of Wnt/β-catenin related genes

**Figure 5:**
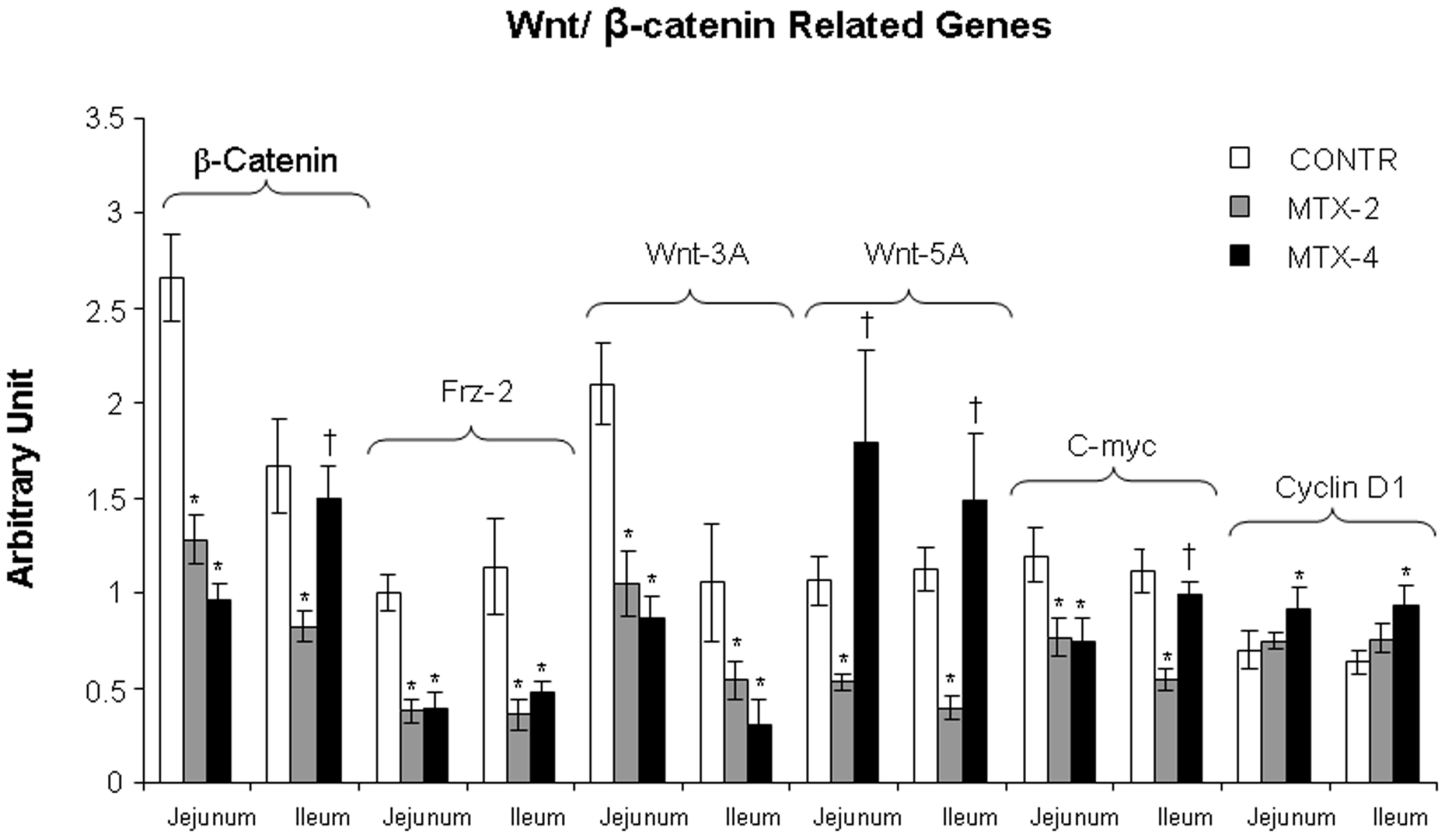
Effect of MTX on Wnt/β-catenin related genes (β-catenin, FRZ-2, Wnt 3A Wnt 5A, c-myc and Cyclin D1 mRNA) expression in gut mucosa following methotrexate induced intestinal damage. Values are mean ± SEM. CONTR- control, MTX-methotrexate. * P<0.05 MTX-2 and MTX-4 vs CONTR rats, † P<0.05 MTX-4 vs MTX-2 rats.

MTX-2 rats (Group B) demonstrated a significant decrease in FRZ-2 (jejunum- two-fold decrease; ileum- two-fold decrease; p<0.05), Wnt 3A (jejunum- two-fold decrease; ileum- two-fold decrease; p<0.05), Wnt 5A (jejunum- two-fold decrease; ileum- three-fold decrease; p<0.05), β-catenin (jejunum- two-fold decrease; ileum- two-fold decrease; p<0.05), c-Myc (jejunum- two-fold decrease; ileum- two-fold decrease; p<0.05) mRNA expression compared to control animals (Group A) (Figure 5). Four days following MTX administration (Group C), rats demonstrated a trend toward a restoration of Wnt/β-catenin signaling especially in ileum. MTX-4 rats showed a significant increase in Wnt 5A (jejunum- three-fold increase; ileum- two-fold increase; p<0.05), β-catenin (ileum- two-fold increase; p<0.05), c-Myc (ileum- two-fold increase; p<0.05) mRNA expression compared to MTX-2 rats (Group B).

#### Western blot

**Figure 6:**
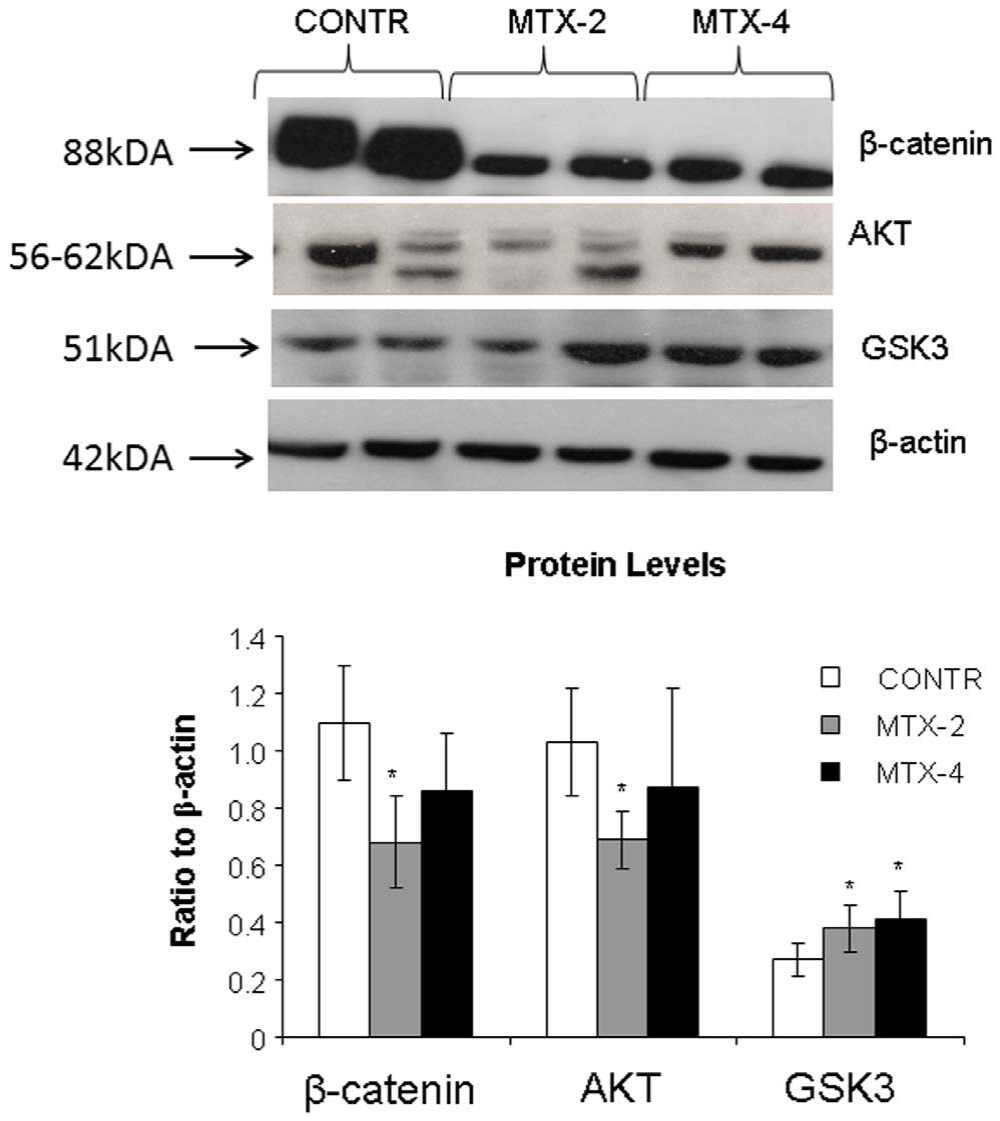
Changes in intestinal mucosal β-catenin, Akt 1/2/3, GSK3 protein levels following methotrexate induced intestinal damage. Values are mean ± SEM. CONTR- control, MTX-methotrexate. * P<0.05 MTX-2 and MTX-4 vs CONTR rats, † P<0.05 MTX-4 vs MTX-2 rats.

Decreased cell proliferation rates in MTX-2 animals (Group B) were accompanied by a significant decrease in β-catenin (two-fold decrease, p<0.05) and Akt (30% decrease, p<0.05) protein levels and a trend toward increase in GSK3 protein levels (NS) compared to control animals (Figure 6). Although MTX-4 rats demonstrated a trend toward increase in β-catenin and Akt protein levels compared to MTX-2 animals, this trend was not statistically significant.

## Discussion

In the current study, the effects of methotrexate on Wnt/β-catenin signaling were investigated both in an in vivo and in vitro model. Pathogenesis of oral mucositis, induced by chemotherapy and radiotherapy, in rats is similar to that seen in humans and has been suggested as a basis of proof-of-concept studies for preclinical testing of therapeutic candidate agents. Gastrointestinal mucositis closely follows the paradigm of an acute mucosal damage phase characterized by inflammation, generation of reactive oxygen species (ROS), producing proteins, such as tumor necrosis factor (TNF), interleukin-1β (IL-1β) and interleukin-6 (IL-6), epithelial cell apoptosis, and ulcerative lesions, followed by a self-healing phase with restoration of mucosal epithelium and barrier function [12], [13].

Extensive experimental evidence suggests that Wnt/β-catenin signaling plays a central role in maintaining the intestinal stem-cell niche and in regulating differentiation of stem cells within the intestinal epithelium toward either enterocytes or one of three secretory cell lineages (Paneth, goblet, or enteroendocrine cells) [14]. β-catenin is a component of the Wnt signaling pathway which was the first to be implicated in the control of the gut stem-cell system. In the absence of a Wnt/Wingless signal, cytoplasmic β-catenin interacts with glycogen synthase kinase-3β (GSK-3β) and the adenomatous polyposis coli (APC) protein, which leads to its phosphorylation in the N-terminal domain and further degradation via the ubiquitin/proteasome pathway [15]. Activation of the Wnt/Wingless pathway inhibits GSK-3β-dependent phosphorylation of β-catenin. Stabilized, hypophosphorylated β-catenin translocates to the nucleus, where it interacts with transcription factors which leads to the increased expression of genes responsible for proliferating activity, such as MYC and CCND1 [16]. In addition to maintaining stem cell activity, Wnt signaling plays an important role in Paneth cell differentiation. Paneth cells reside at the base of the crypt, where Wnt protein is plentiful, and Wnt signaling drives their differentiation [17].

The role of Wnt/β-catenin in development chemotherapy-induced mucositis and in recovery following gut damage in unknown. A recent study has shown that Wnt modulator, RSpo1 significantly reduces both chemotherapy and radiotherapy-induced damage to the oral mucosa by amplifying the Wnt/β-catenin signaling and subsequently triggering epithelial cell growth to accelerate mucosal healing in mice [18]. In the current study, we thus assessed the time-dependent effect of methotrexate on Wnt/β-catenin signaling in a rat model of chemotherapy-induced intestinal mucositis.

The *in-vitro* study has shown that treatment with methotrexate dramatically inhibited early cell proliferation measured by BrdU incorporation into cellular DNA. Although late cell proliferation rates increased significantly compared to early proliferation, it was still 36-fold lower in comparison with control cells with vehicle alone. In addition, MTX resulted in marked induction of cell apoptosis. Treatment with MTX resulted in a significant six-fold increase in early apoptosis and four-fold increase in late apoptosis compared to Caco-2 untreated cells. These data suggest that MTX caused more significant early damage and that there was a trend toward damage repairing during the late stages. The anti-proliferating and pro-apoptotic effects of MTX on epithelial cells was accompanied by a significant decrease in target gene c-Myc compared to untreated cells. However, two days after exposure to MTX Caco-2 cells demonstarted a significantly higher levels of β-catenin mRNA levels over corresponding control cells with vehicle alone suggesting initiating cell recovery. *The in-vivo* experiment has demonstrated that treatment with methotrexate resulted in clear cut intestinal damage. This conclusion is supported by the observed increase in intestinal injury score compared to control animals. MTX rats also showed severe villous atrophy, epithelial flattening, and extensive crypt loss. In addition, treatment with MTX resulted in significant mucosal hypoplasia. This damaging effect was maximal 2 days after MTX administration. A decrease in bowel and mucosal weight, mucosal DNA and protein, and in villus height support this conclusion. Parallel decreases in mucosal DNA and protein indicate that the smaller mucosal mass of MTX-2 animals can be attributed to cellular hypoplasia. Histologically, villus height decreased in response to MTX administration, suggesting decreased absorptive surface area; however, crypt depth decreased non-significantly. Analysis of epithelial proliferation two days after MTX administration, using BrdU incorporation as a marker, demonstrated an inhibition of DNA synthesis in the epithelium of the entire small intestine. In addition, the proliferative zone in MTX-rats moved progressively upwards in the crypts toward the crypt-villus junction. The mechanism responsible for this effect is poorly understood. Cell loss in the small intestine MTX-induced mucositis is mainly regulated by programmed cell death. Our results show that apoptosis increased significantly after 2 days, but reached a maximal rates after 4 days. Despite high rates of programmed cell death, damaged intestine of MTX-4 rats exhibited the signs of initial recovery. MTX-4 rats demonstrated a trend toward increase in bowel and mucosal weight, and mucosal protein compared to MTX-2 animals. However, microscopically no improvement in villus height and crypt depth was observed. MTX-4 animals also showed a trend toward an increase in cell proliferation rates compared to MTX-2 rats; however, this trend was not statistically significant.

The overall effect of biologically active Wnt proteins appears to involve a combination of signaling through the cell-specific activation of various target genes in a tissue-dependent manner. A few previous studies have examined changes in Wnt gene expression in intestine following chemotherapy-induced mucositis. In this experiment, the expression levels of several candidate canonical class Wnt transcripts were found to be significantly decreased two days after MTX administration. Conversely, the expression level of FRZ, Wnt 3A, Wnt5A, and C-myc were suppressed following MTX administration. In previously published reports, Wnt1, Wnt2, Wnt2B, Wnt3, Wnt3A, Wnt7B, Wnt8A have shown a preferential ability to signal through β-catenin [19]. Similarly Wnt4, Wnt 5A, and Wnt11 were reported by various researchers to signal by alternative intracellular signaling mechanisms, including Ca2+-mediated pathways or the c-Jun N-terminal kinase cascade [20]. In most systems tested, the various Wnts that signal through β-catenin appear to promote cell proliferation, whereas Wnt proteins that signal through alternative or noncanonical pathways seem to promote differentiation and oppose proliferation and cellular multipotency. Specifically, Wnt5A was shown to promote differentiation in a variety of tissue types while opposing the proliferative effects of canonical Wnts, including Wnt1 and Wnt3A [21]. In the current study, a significant increase in Wnt 5A and β-catenin was observed four days after MTX administration when compared to MTX-2 animals. It should be emphasized that alternative intracellular signaling mechanisms (Ca2+-mediated pathways or the c-Jun N-terminal kinase cascade) are involved in the adaptive process in MTX-4 rats in order to promote cell differentiation.

Our data lead us to speculate that canonical Wnt signaling and β-catenin activation are specific mechanisms used by intestinal stem cells for transit through the cell cycle during intestinal recovery. Whether this is a promoting effect through activation of cell-cycle progression genes such as cyclin kinases or a permissive effect through repression of cell cycle arrest genes such as cyclin kinase inhibitors is not known, but these are possible explanations for the differing roles of β-catenin during various mechanisms of intestinal regeneration. One of the earliest Wnt/β-catenin target genes identified in mammalian cells was the c-myc proto-oncogene, hereafter referred to as *Myc* [22]. *Myc* is a transcription factor that regulates expression of a battery of target genes that control cellular proliferation, metabolism, and apoptosis [23]. Given our findings that β-catenin localized to proliferating intestinal stem cells, and evidence of target gene activation (c-Myc) on day 4, we speculate that one or a number of the Wnt family members we have identified use the canonical pathway in the intestine during the generative response. Moreover, because the various Wnt transcripts localize within the ADP themselves, this would suggest there may be at least partial autocrine regulation of the Wnt signaling axis in the intestine.

These findings provide new evidence for a critical role of Wnt signaling in regulating the regenerative response of the gut through intestinal stem cell proliferation. Further studies as to the precise role of each of the Wnt family members and the combination signaling that regulates the overall biologic effect will be enlightening. As our knowledge of the overall importance of Wnt/β-catenin signaling in both normal and diseased intestine is increases, findings such as those presented here of proto-oncogenic Wnt/β-catenin activation in the gut during MTX-induced injury may have profound implications for intestinal regeneration.
